# Generation of ribosome imprinted polymers for sensitive detection of translational responses

**DOI:** 10.1038/s41598-017-06970-x

**Published:** 2017-07-26

**Authors:** Helen A. King, Hazim F. El-Sharif, Ana M. Matia-González, Valentina Iadevaia, Adeola Fowotade, Subrayal M. Reddy, André P. Gerber

**Affiliations:** 1 Department of Microbial Sciences, Faculty of Health and Medical Sciences, University of Surrey, Guildford, Surrey, GU2 7XH United Kingdom; 2 Department of Chemistry, Faculty of Engineering and Physical Sciences, University of Surrey, Guildford, Surrey, GU2 7XH United Kingdom; 3 Chemistry Division, School of Physical Sciences and Computing, JB Firth Building, University of Central Lancashire, Preston, Lancashire PR1 2HE United Kingdom

## Abstract

Whilst the profiling of the transcriptome and proteome even of single-cells becomes feasible, the analysis of the translatome, which refers to all messenger RNAs (mRNAs) engaged with ribosomes for protein synthesis, is still an elaborate procedure requiring millions of cells. Herein, we report the generation and use of “smart materials”, namely molecularly imprinted polymers (MIPs) to facilitate the isolation of ribosomes and translated mRNAs from merely 1,000 cells. In particular, we show that a hydrogel-based ribosome imprinted polymer could recover ribosomes and associated mRNAs from human, simian and mice cellular extracts, but did not selectively enrich yeast ribosomes, thereby demonstrating selectivity. Furthermore, ribosome imprinted polymers enabled the sensitive measurement of an mRNA translational regulatory event, requiring 1,000-fold less cells than current methodologies. These results provide first evidence for the suitability of MIPs to selectively recover ribonucleoprotein complexes such as ribosomes, founding a novel means for sensitive detection of gene regulation.

## Introduction

Translation plays a central role in the regulation of gene expression and its deregulation is considered to contribute to a variety of pathological conditions including cancer^1, 2^. The ribosome comprises the core macromolecular machine for translation; responsible for decoding the genetic information contained in messenger RNA (mRNA) and catalysing the formation of peptide bonds for protein synthesis. Ribosomes are highly conserved across species: in eukaryotes, they are comprised of four ribosomal RNAs (rRNAs) and about 80 ribosomal proteins (RPs), which are shared by two subunits referred to as the large (60S) and the small (40S) ribosomal subunit^3, 4^. The major differences of ribosomes across eukaryotes relate to rRNA expansion segments that have evolved as additions to rRNAs without perturbing the pre-existing core^5^.

Recent studies revealed that the steady-state mRNA levels in cells (transcriptome) only fairly correlate with protein levels (proteome)^6^, indicating that post-transcriptional regulatory events such as mRNA translation, play a major role in gene expression^7–9^. For instance, it has been estimated that about 40% of protein levels are determined at the level of mRNA translation in immortalized mouse fibroblasts^9^. Translational control can be accomplished by the interplay between *trans*-acting factors (*e.g*. components of the translation machinery, RNA-binding proteins or microRNAs) and *cis*-acting elements, such RNA sequences or structures in the mRNAs^10, 11^. The analysis of the translatome, which refers to all mRNAs recruited to ribosomes for protein synthesis^12^, could therefore uncover important regulatory events for maintaining proper cell homeostasis and the etiology of diseases^1, 13^.

Different techniques are currently used to measure mRNA translation (reviewed in ref. 14). Classical polysomal profiling relies on the fractionation of cycloheximide (CHX; an inhibitor of translation elongation) treated cellular extracts through a linear sucrose density gradient by ultracentrifugation, thereby separating mRNAs based on the number of bound ribosomes which is indicative of translational activity^15^. More recent ribosomal profiling approaches include the digestion of mRNAs with RNases, enabling the detection of the position of the ribosome along the mRNA at codon-resolution by deep sequencing of ribosome protected footprints (RPFs)^16, 17^. Beside the measurements of translation rates of mRNAs at a global level, ribosome profiling also identified *cis*-acting translational control elements, such as upstream open reading frames and alternative start codons; and it suggested the translation of numerous micropeptides, some of them encoded in long non-coding RNAs (lncRNAs)^17^. Nevertheless, polysomal as well as ribosomal profiling involve an elaborate procedure (*e.g*. ultracentrifugation) that requires millions of cells to obtain sufficient amounts of RNA for further analysis, limiting their application to cultured cells or well-accessible tissues. An alternative approach that was introduced to access gene expression of specialised cells involves the affinity-isolation of ribosomes and associated mRNAs via tagged RPs^18–20^. However, these translating ribosome affinity purification (RAP/TRAP) techniques require genetic manipulation of cells or organisms for integration of an affinity tag into a particular RP^14^.

**Figure 1:**
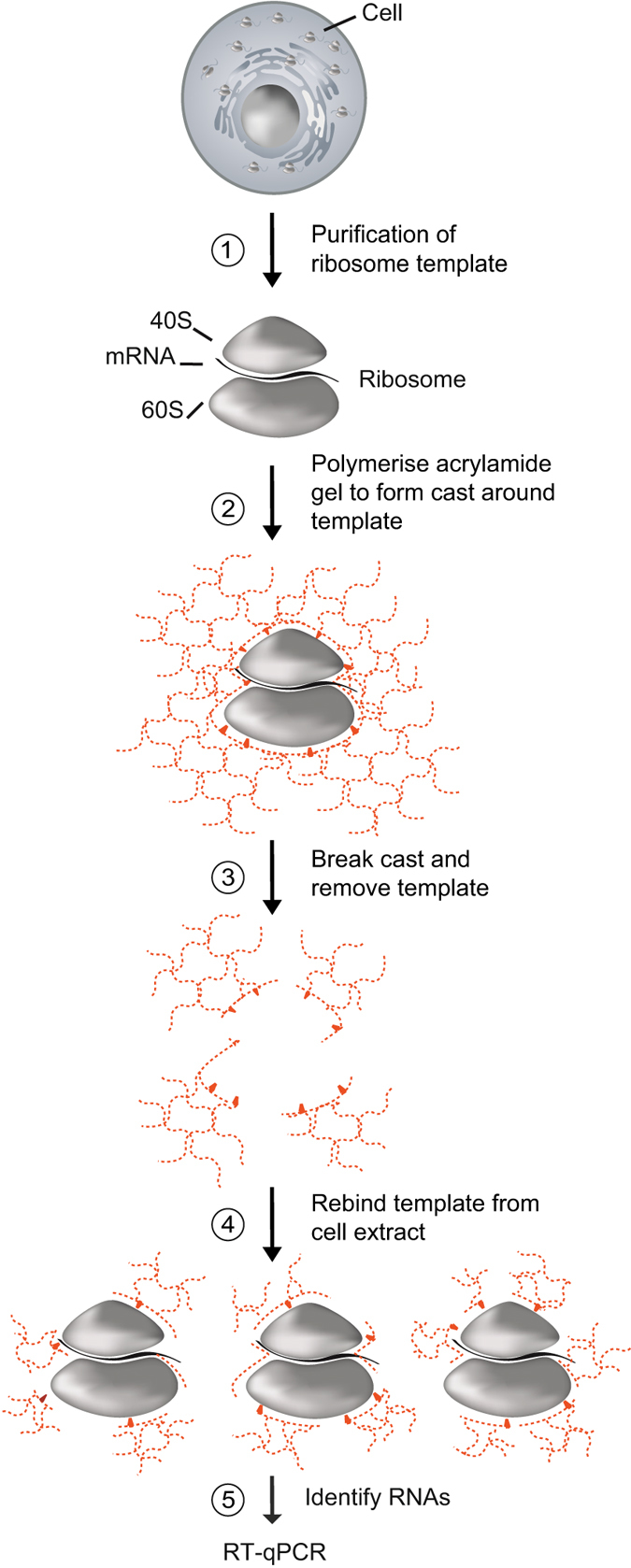
Schematic overview of R-MIP preparation. First, ribosomes are isolated from HeLa cell cytoplasmic extract using a sucrose cushion. Second, the ribosome template is combined with a mixture of acrylamide (AA) and *N*,*N*′-methylenebisacrylamide (MBAm) monomers, and polymerisation is induced under gaseous nitrogen upon addition of the initiator ammonium persulfate (APS) and the catalyst *N*,*N*,*N*′,*N*′-tetramethylethylenediamine (TEMED). Third, the hydrogel is granulated by passing through a sieve mesh, and the ribosome template is removed from the MIP. This results in a slurry of heterogeneous PAA fragments, with cavities possessing the potential to recognise more template, based both upon three dimensional structure and direct interactions between the template and chemical groups on the surfaces of the cavities. Fourth, MIPs are combined with cellular extracts to capture ribosomes and associated mRNAs. Fifth, ribosome-associated mRNAs are isolated from the MIP for further analysis, such as reverse transcription (RT)-quantitative PCR (qPCR).

Molecularly imprinted polymers (MIPs) are so-called “smart materials” with potential to replace antibodies in the future^21^. The concept of the MIP technology relies on the formation of a synthetic polymer around a template molecule, which after removal, leaves behind an idealised “imprint”; cavities within the polymer matrix, which can specifically recognise the template molecule (Fig. 1)^22–26^. MIPs found early application for solid phase extraction of drugs and pesticides^27^, and more recently for bioanalyte detection and as biosensors^28, 29^. In particular, hydrogel MIPs based on polyacrylamide (PAA) and derivatives thereof were realised for selective recognition of bio-macromolecules, including, but not limited to, small regularly shaped globular proteins such as bovine serum albumin (BSA)^30, 31^ or haemoglobin^32, 33^. Protein-based MIPs were also tested as nucleates for protein crystallisation^34^.

In an attempt to resolve the current limitations in translatome profiling, we applied MIP-based “smart materials”, namely, the PAA-based bulk MIP technology, for the isolation of ribosomes and the associated translating mRNAs from cell extracts equivalent to 1,000 cells. Using purified human ribosomes as a template, we were able to imprint and elute the ribosome from the PAA-based MIP, thus generating a heterogeneous population of MIP particles. These ribosome imprinted polymers, which we will henceforth refer to as ribosome-MIPs (R-MIPs), were then used to recover ribosomes from human, simian and mouse cellular extracts; but they could not selectively enrich ribosomes from yeast extracts, showing specificity towards closely related template ribosomes. Furthermore, we could recapitulate the serum-induced translational regulation of *RPS6* mRNAs in 1,000 human cells through application of R-MIPs. In conclusion, our results provide first proof-of-concept for the suitability of “smart materials” to monitor changes in gene expression.

## Results

To capture ribosomes and translated mRNAs from few cells, we adapted and further developed a bulk molecular imprinting technique previously applied to small molecules, globular proteins as well as regularly shaped viruses (Fig. 1)^22–26, 35^. The procedure included: 1) the preparation of human active ribosomes; 2) the imprinting of these ribosomes in a PAA hydrogel by polymerisation of monomers with a cross-linker to form a cast; 3) the removal of the ribosome template from the MIP; 4) the rebinding of translating ribosomes from extracts to R-MIPs; and 5) the isolation and amplification of ribosome bound RNAs by reverse transcription (RT) polymerase chain reaction (PCR). Importantly, a non-imprinted polymer (NIP) was prepared in parallel to control for non-specific binding of ribosomes/RNA to the hydrogel. Therefore, the polymerisation of acrylamide (AA) was performed with buffers lacking the ribosome template. In the following, we describe the optimisation and validation of each step of the procedure.

### Preparation of template ribosomes for imprinting (step 1)

**Figure 2:**
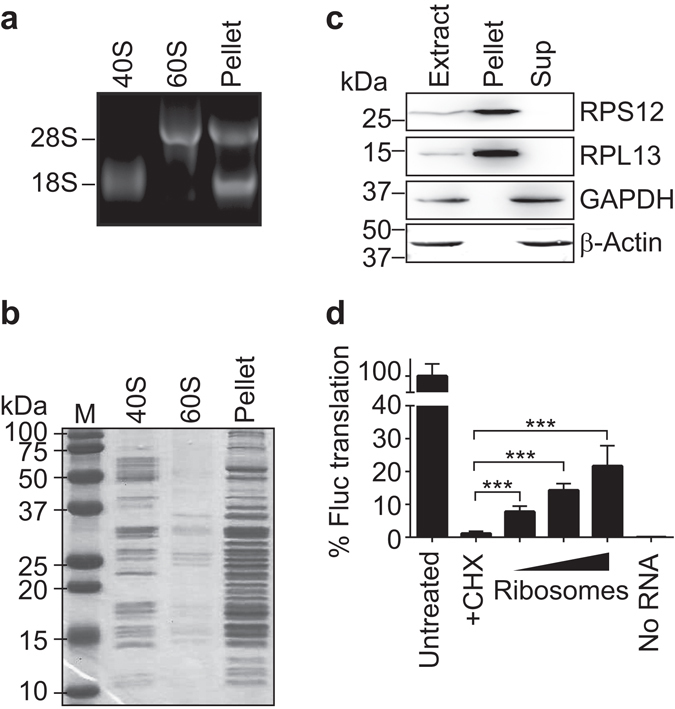
Preparation of translationally active ribosomal template. (**a**) RNA extracted from the purified 40S and 60S subunits and whole ribosome pellet were separated on an agarose gel; the position of 28S and 18S rRNA are indicated. (**b**) Colloidal coomassie stained 15% PAA gel for visualisation of proteins contained in the ribosome pellet and purified 40S and 60S ribosomal subunits. A marker (M) with molecular weights in kilodaltons (kDa) is shown to the left. (**c**) Immunoblot analysis with specific antibodies detecting RPS12, RPL13 (RPs as positive control), GAPDH and β-actin (non-RPs as negative control). Lanes refer to the following: Extract, total cytoplasmic extract; Pellet, ribosome pellet; Sup, supernatant of the ribosome pellet. Images of uncropped gels and blots are shown in the Supplementary Fig. S1. (**d**) *In vitro* translation assays in rabbit reticulocyte lysate. CHX was added to lysates to inhibit translational activity, followed by supplementation of the lysate with increasing amounts of purified human ribosomes (2 μg, 4 μg, and 8 μg). Height of the bars indicates relative bioluminescence of translated firefly Luc protein (Fluc) compared to untreated lysates (100%). A sample without RNA (No RNA) was added as a control to measure background bioluminescence of untreated lysates. Error bars represent standard deviation (SD), n = 3. ***P < 0.005, two-tailed student’s *t*-test with equal variance.

We purified ribosomes from HeLa cell derived cytoplasmic extracts by classical sucrose density fractionation (see Methods). In brief, the extracts were supplied in high salt buffer to remove ribosome-associated proteins and further subjected to mild RNase treatment to separate polysomes into monosomes without substantial degradation of rRNA. The extract was then centrifuged through a sucrose cushion to enrich for whole ribosomes in the pellet, analogous to procedures used for ribosomal profiling^16^. For comparison, we also purified 40S and 60S ribosomal subunits from extracts treated with puromycin, a compound that dissociates translating ribosomes. As expected, total RNA isolated from the pellet (whole ribosome fraction) contained 18S and 28S rRNAs, and the corresponding rRNA species were present in purified 40S and 60S ribosomal subunits, respectively (Fig. 2a). Likewise, a subset of the cellular proteins was selectively enriched in 40S, 60S, and whole ribosome preparations (Fig. 2b). Further immunoblot analysis revealed that RPS12, a RP of the 40S subunit, as well as RPL13 of the 60S subunit were present in the ribosomal pellet, whereas glyceraldehyde 3-phosphate dehydrogenase (GAPDH) and β-actin, which are abundant cytoplasmic proteins, were not detectable in the pellet and remained in the supernatant (Fig. 2c, images of uncropped gels and blots are shown in the Supplementary Fig. S1).

We next tested whether the purified ribosomes remained active, as we reasoned that this might be crucial to develop a MIP for capturing translating ribosomes. Therefore, we used a commercial rabbit reticulocyte lysate translation assay to measure the translation of *in vitro* transcribed luciferase (Luc) reporter mRNAs. Thereby, we first inhibited translation in the rabbit lysate with CHX and then added increasing amounts of purified human ribosomes (Fig. 2d, for details see Methods). To roughly estimate the activity of our purified human ribosomes, we further compared the abundance of selected RPs in the reticulocyte lysate with that of added purified human ribosomes (Supplementary Fig. S2). The highest amount of purified human ribosomes added to the assay (8 μg) was equivalent to ~60% of rabbit ribosomes present in the lysate. It restored the translational activity to 22% (±6.2%), which allowed us to estimate that equal amounts of purified human ribosomes could recover ~36% of the translational activity of rabbit lysates (Fig. 2d). Although this assay can only give a rough estimate of the translation activity due to various factors, *e.g*. complementation of rabbit ribosomes with purified human ones, we were nevertheless satisfied that our purified ribosomes retained substantial activity.

### Optimising the imprinting procedure (step 2 and granulation)

As the imprinting process subjects the template ribosome to chemical and physical stresses, we first investigated whether ribosomes remained intact upon imprinting. Therefore, a mixture of AA monomer and *N*,*N*′-methylenebisacrylamide (MBAm) cross-linker was polymerised in the presence of purified ribosomes, and the so-formed hydrogel was granulated by passing through a sieve and washed extensively with physiological buffers (Fig. 1; Methods). To release the imprinted proteins and RNAs for further analysis, the granulated MIPs containing the imprinted ribosome template (referred to as Rt-MIP) were physically disrupted with glass beads (Fig. 1, step 5).

**Figure 3:**
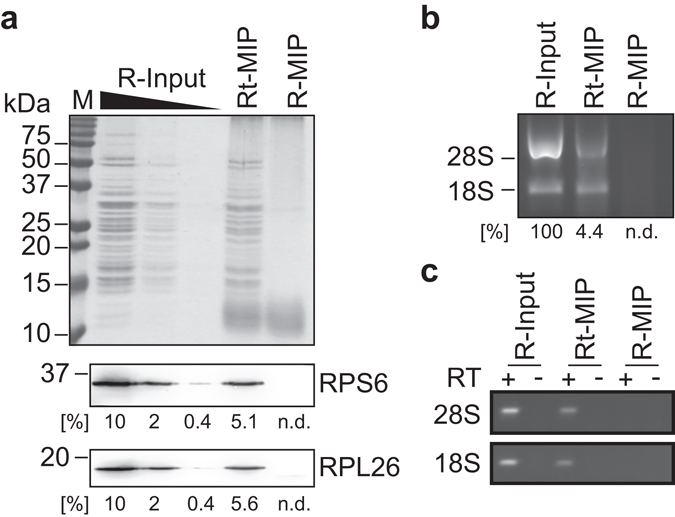
Imprinting and removal of template ribosomes from MIPs. (**a**) Coomassie stained PAA gel showing a titration of the purified ribosomes used for imprinting (R-input), and the content of sieved and granulated MIPs before (Rt-MIPs) and after (R-MIP) elution of the ribosome template. An immunoblot for detection of RPS6 (protein of the 40S subunit), and RPL26 (protein of the 60S subunit) is shown at the bottom. A quantification depicting the fraction of RPs compared to R-input (100%) is indicated at the bottom of each lane; no signal was detected for R-MIPs (n.d.). A molecular weight marker (M) is indicated to the left. (**b**) Agarose gels showing the same set of samples as in (**a**) to visualize 18S and 28S rRNAs as indicated. Bands corresponding to 18S and 28S rRNAs were quantified with ImageJ, and the averaged recovery (%) is indicated below the panel. No signal could be detected for R-MIPs (n.d.). Images of uncropped gels and blots are shown in the Supplementary Fig. S1. (**c**) RT-PCR for detection of indicated rRNA species. Control reactions without RT (−) are shown next to samples performed with RT (+).

We could not observe the presence of degraded proteins or rRNA in the Rt-MIP as compared to the original ribosomes used for imprinting, suggesting that ribosomes remained intact during imprinting and physical extraction (Fig. 3). However, quantification of RPs and rRNA indicated that only about 5% of the initially applied ribosomes (R-Input) were detectable in the Rt-MIP (~37.5 μg/ml of ribosomes; Fig. 3a,b). The apparent low recovery might have various reasons but could indicate that extensive washing of the granulated Rt-MIPs with physiological buffers removes a large fraction of weakly bound ribosomes. This is not unusual when preparing bulk MIPs as the granulation of MIP through sieving leads to a mixture of some MIP particles comprising highly selective cavities but a majority of material being redundant unselective particles, devoid of highly template-specific cavities. Nonetheless, we were confident that we were producing an imprint of non-degraded, and therefore likely biologically representative ribosomes with substantial affinity to the MIP.

### Removal of template ribosomes to obtain the R-MIP (step 3)

We next tested for efficient removal of the remaining ribosome from the granulated Rt-MIP (Fig. 1, step 3). This is important as any remaining ribosomes in the matrix could impinge re-binding of ribosomes and obscure further analysis due to remaining contaminating RNAs. We observed that standard procedures, such as sodium dodecyl sulfate (SDS) and high-salt buffers^32^, were inappropriate to completely remove ribosomes from the Rt-MIP (unpublished results). Therefore, we established an alternative procedure to target RNAs and proteins more specifically: firstly, by subjecting the granulated Rt-MIPs to alkaline hydrolysis with sodium hydroxide (NaOH) to degrade RNA; and secondly, by treatment with proteinase K to degrade proteins. The MIP was finally washed with physiological buffers to remove remaining RNA or proteins and/or fragments thereof, and its contents were physically extracted for further analysis. We found that this protocol enabled the efficient removal of RPs and rRNAs from the Rt-MIP as analysed by PAA gels, immunoblot analysis of RPs and RT-PCR of 18S and 28S rRNAs (Fig. 3, compare “Rt-MIP” *vs*. “R-MIP”). The quantification by RT-qPCR revealed that 99.89% (SD ± 0.06%, n = 6) of 18S rRNA could be removed from the MIP (~42 ng of remaining rRNA per 50 μl R-MIP), which is superior to previously reported removal efficiencies of template proteins (*e.g*. 90%^32^). We thus concluded that ribosomes could be effectively removed from MIPs, although a low fraction of peptide/RNA fragments could have persisted in the R-MIP.

### Selective binding of ribosomes to human R-MIPs (step 4)

We reasoned that our R-MIPs could be used to capture ribosomes and associated mRNAs from cell extracts derived from human and closely related species, as ribosomes are highly conserved^5^. To test the selectivity of R-MIPs in line with our original aim to develop a method for isolation of ribosomes from few cells, we applied cellular extracts corresponding to ~1,000 cells from human, simian, mouse, and yeast cells to the MIP or NIP, respectively. The captured ribosomes and associated mRNAs were then recovered from the hydrogels after extensive washing. Specific mRNAs expressed in the respective organisms were amplified by RT-qPCR and quantified. Of note, any technical bias imposed during RNA isolation and RT-PCR was controlled by the addition of exogenous spiked-in RNA (*LysA*) to the samples prior to RNA isolation.

**Figure 4:**
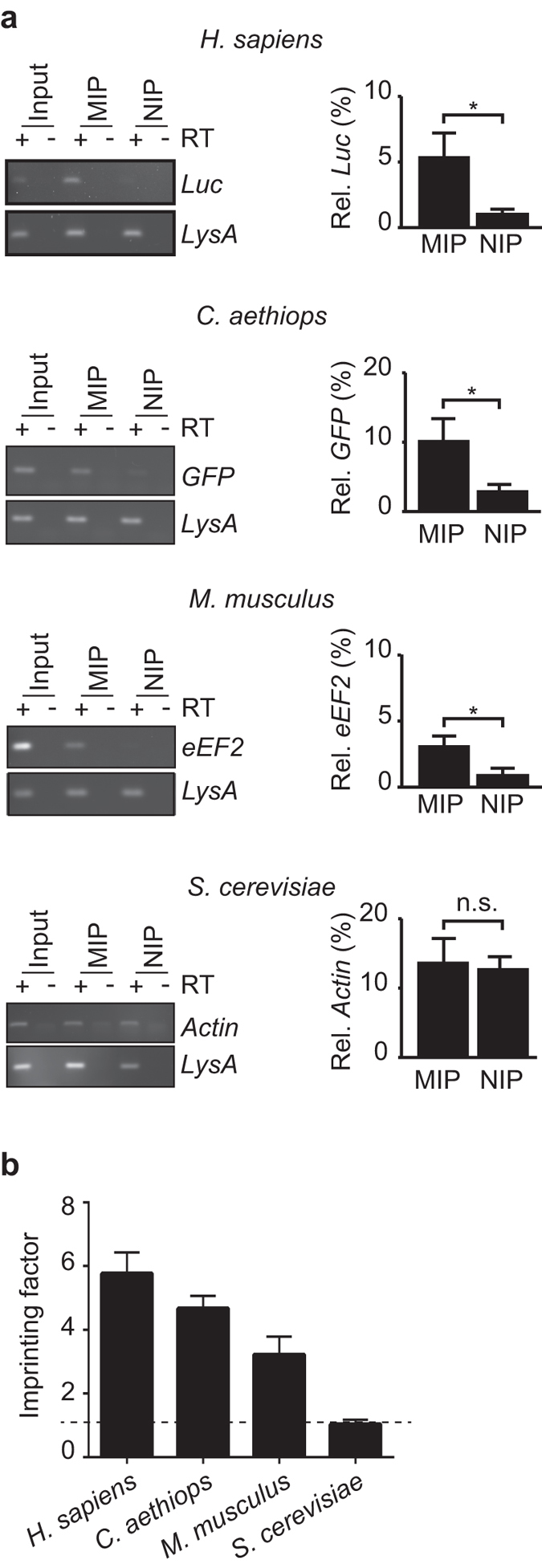
Human R-MIPs can be used to recover ribosome-associated mRNAs from cellular extracts of closely related eukaryotic species. (**a**) Detection of the indicated mRNAs from the specified species in extracts, MIPs and NIPs. RT-PCR products were visualised on agarose gels shown to the left. *LysA* is a spiked-in control used for normalisation. Images of uncropped gels and blots are shown in the Supplementary Fig. S1. The chart to the right shows relative recovery of indicated mRNAs with MIPs or NIPs as compared to the input extract (100%). RNA was quantified by RT-qPCR with the ΔΔCt method and normalised to *LysA* (see Methods). Standard error of means (SEM) are shown as bars; *H. sapiens*, n = 5, Luc reporter (pGL3) in HeLa cells; *C. aethiops*, n = 6, GFP reporter in VK219 cells; *M. musculus*, n = 5, endogenous eEF2 mRNA in C8-D1A cells; *S. cerevisiae*, n = 3, actin mRNA in yeast. *P < 0.05, two-tailed homoscedastic student’s *t*-test. (**b**) Imprinting factor (mean  ± SEM) related to specified species ordered according to the evolutionary distance from human. The dotted line marks an IF of 1, indicating no preferential binding to MIP compared to NIP.

To exclude the possibility of measuring any residual mRNAs originating from the human template ribosomes (see above), the R-MIPs were reloaded with extracts derived from transfected HeLa cells that constitutively expressed firefly luciferase (Luc). In this setting, any signal of the reporter RNA detected in the MIP must originate from the re-loaded ribosomes as the reporter was not present during MIP preparation. Although the recovery of *Luc* mRNA was relatively low compared to total RNA levels obtained from 1,000 cells, which is in line with the previously observed low recovery of ribosomes upon imprinting possibly due to a large fraction of low affinity binding sites on the R-MIP; we found that the *Luc* reporter mRNA was significantly enriched with R-MIPs compared to the negative control NIPs (*P* < 0.05; n = 5, two-tailed homoscedastic *t*-test) (Fig. 4a). The imprinting factor (IF; corresponding to the ratio of the amounts of *Luc* mRNA recovered from the MIP compared to the NIP) was 5.77 (SE ± 0.66), which is in line or above previously reported IFs for protein MIPs^36, 37^ (Fig. 4b). Likewise, a viral GFP reporter expressed in simian *Chlorocebus aethiops* VK219 cells^38^, as well as endogenously expressed *eEF2* and *GAPDH* mRNAs from the mouse neuronal cell line C8-D1A, were enriched with R-MIPs compared to the NIP controls (IF_GFP_ = 4.67 ± 0.39; IF_eEF2 = _3.22 ± 0.57, IF_GAPDH_ = 3.28 ± 0.52, respectively), indicating the enrichment of corresponding ribosomes from these species (Fig. 4, Supplementary Fig. S3). In contrast, the direct application of 6 ng of mouse total RNA (corresponding to the amount of total RNA from 1,000 cells) to R-MIPs showed no specific recovery of *eEF2* mRNA (0.28% ± 0.25 of the initially applied mRNAs), demonstrating only minor direct binding of RNAs to the hydrogel MIP (Supplementary Fig. S4). Furthermore, the mainly untranslated *CHOP* mRNA^39^ was detectable in the total RNA fraction isolated from human and avian cell extracts, but it was not at quantifiable levels with R-MIPs and NIPs (data not shown). Possibly more revealing, the application of cell extract from *S. cerevisiae* showed no significant recovery of the well-expressed endogenous actin (*ACT1*) and glyceraldehyde-3-phosphate dehydrogenase (*TDH1*) mRNAs with R-MIPs compared to NIPs (IF_ACT1_ = 1.04 ± 0.14; IF_TDH1_ = 2.19 ± 0.62) (Fig. 4, Supplementary Fig. S3). Thus, although we cannot completely rule-out the possibility for some association of other macromolecular complexes to R-MIPs, these results suggest that we were selectively capturing ribosomes using specific MIP cavities; moreover since human R-MIP could be used to recover mRNAs of closely related species (simian and mice) but not of a distantly related eukaryote, such as yeast. Of note, this finding correlates with the evolutionary conservation and intrinsically owned structural similarities of ribosomes across these species.

### Measuring the translational regulation of a specific mRNA

**Figure 5:**
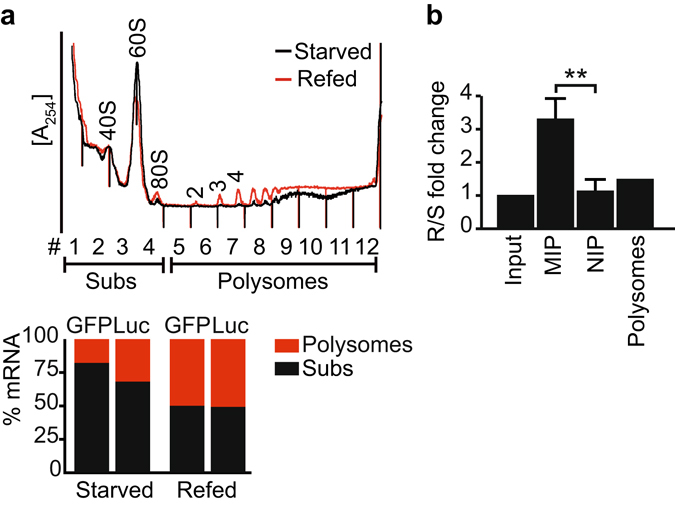
Detection of the translational response of an exogenous reporter mRNA in 1,000 human cells with R-MIPs. (**a**) The absorbance profile at 254 nm across a sucrose gradient is shown at the top, and the positions of the 40S and 60S ribosomal subunits, 80S monosomes, and polysomes are indicated. The black line represents a profile of co-transfected HeLa cells grown in serum-starved conditions (starved); the red line upon stimulation of the cells with serum (refed). The bar chart depicts the distribution of *RPS6-GFP* (left) and *Luc* control (right) mRNAs in subpolysomes (fractions 1–4) and polysomes (fractions 5–12) of starved and refed cells. RT-PCR data was normalised to a spiked-control RNA (*LysA*) added to each fraction prior to RNA isolation to adjust for technical variation during RNA isolation. (**b**) Bar chart depicting the changes of *RPS6-GFP* relative to *Luc* mRNA in refed (R) *vs*. starved (S) conditions in MIP/NIP eluates and in polysomes. RT-qPCR data was normalised to the respective mRNA levels in cell extracts (input) to adjust for variation in the transcriptome upon treatment of cells (see Methods). The change in polysomes was analysed upon pooling fractions 5–12 from the sucrose gradient shown in *a*. Error bars represent SEM, n = 3. **P < 0.01, two-tailed homoscedastic student’s *t*-test.

We finally wondered whether ribosomal MIPs could be used to measure a specific translational response in as little as 1,000 human cells. Therefore, we co-transfected HeLa cells with two reporter plasmids: pGL3, which constitutively expresses firefly Luc and served as a reference; and pRPS6-GFP, which expresses the coding sequence (CDS) of GFP flanked by the 5′ and 3′ UTR sequences of human *RPS6* ^40^. *RPS6* belongs to a class of mRNAs bearing a terminal oligo-pyrimidine tract (TOP) sequence (which is usually a cytosine followed by a tract of 6–10 pyrimidines) in the 5′ UTR^41, 42^. TOP mRNAs code for RPs, translation elongation factors and some initiation factors, whose translation is particularly regulated in a growth-dependent manner. In actively growing cells, TOP mRNAs are preferentially associated with polysomes for translation; whereas in quiescent or growth-arrested cells, they are sequestered into inactive mRNA ribonucleoprotein (mRNP) complexes^41, 42^. It was shown that the fraction of *RPS6*-driven GFP reporter RNAs in polysomes increased ~1.75-fold upon stimulation of starved HEK293 cells with serum^40^. We thus recapitulated these experiments in HeLa cells that were co-transfected with the two reporter plasmids. Therefore, cells were serum-starved for 2 h (“starved cells”) and then stimulated by addition of 10% serum for 2 h (“refed cells”; see Methods). We then performed sucrose density fractionation of CHX-treated cells, and monitored the distribution of *Luc* and *RPS6-GFP* reporter mRNAs in subpolysomal and polysomal fractions with RT-PCR. As expected, global translation was activated in the refed cells as evident from the increased amplitude of the recorded polysomes (Fig. 5a). Furthermore, *RPS6-GFP* as well as *Luc* mRNAs became increasingly associated with polysomes in refed cells. Importantly, this redistribution was markedly more pronounced for the TOP-sequence bearing *RPS6-GFP* reporter as compared to non-TOP *Luc* mRNAs (~1.75-fold), likely reflecting the translational control inferred on TOP mRNAs as previously reported under these conditions^40^.

To test whether R-MIPs could be used to detect the translational activation of TOP *RPS6-GFP* mRNAs as compared to the non-TOP *Luc* mRNAs, the extracts equivalent to ~1,000 starved and refed cells were loaded onto the R-MIPs and control NIPs, and the ribosome-associated RNAs were subsequently isolated from the MIP and NIP after addition of an externally added reference RNA (*LysA*). The levels of the reporter mRNAs and *LysA* were then measured with RT-qPCR in the MIP/NIP eluates and in total RNA samples isolated from extracts (input). Importantly, the relative changes in reporter expression in refed *vs*. starved cells could then be calculated by taking into account for changes in total mRNA levels upon treatment of cells (=input), thereby adjusting for changes in the transcriptome (see Methods). For comparison, likewise analysis was also conducted with pooled fractions 5–12 of polysomes. This analysis revealed that the *RPS6-GFP* mRNA was on average 3.3-fold (SEM ± 0.65, n = 3) enriched in ribosomes isolated with R-MIPs from refed as compared to starved cells (Fig. 5b). Importantly, the negative control NIP did not capture any more *RPS6-GFP*, supporting our previous observations that the measured changes are due to specific interactions with the bespoke hydrogel (1.13-fold, SEM ± 0.33). The likewise analysis with pooled fractions 5–12 of polysomes revealed a 1.45-fold net increase of *RPS6-GFP* compared to *Luc* mRNAs in refed *vs*. starved cells (Fig. 5b). Hence, more pronounced changes of *RPS6-GFP* were seen using R-MIPs as compared to polysomes. This could be due to the different amounts of cell extracts and RNAs used for analysis (1,000 fold difference), and the different experimental set-ups as R-MIPs likely recover ribosome associated mRNAs irrespective of the number of ribosomes bound to it. Nevertheless, both approaches recapitulated a pronounced translational response of a TOP mRNA as compared to a non-TOP mRNA under the chosen experimental conditions. Therefore, it adds further evidence that R-MIPs can be used to specifically capture ribosome-bound mRNAs and are suitable for the detection of translational regulatory events imposed by changing external conditions.

## Discussion

We describe the first implementation of “smart materials”, namely MIPs, to selectively enrich large cellular macromolecular complexes, such as the ribosome. The MIP-captured ribosomes then allowed the detection of associated mRNAs and corresponding translational responses, as exemplified by the translational regulation of TOP-mRNAs upon serum stimulation of cells. Importantly, application of R-MIPs enabled detection of mRNA translation in extracts derived from 1,000 cells, which is in contrast to current requirements for millions of cells using polysomal and/or ribosomal profiling. Taken together, these results provide proof-of-concept for application of MIPs as a novel means for sensitive detection of gene expression in general, and of translation in particular, and it may eventually open the door for investigation of translation in specialised tissues/cells, which is not feasible with current methodologies. Besides the capture of small globular proteins, the largest molecules (~40 MDa) so far isolated utilising the MIP technology are viruses, such as tobacco mosaic virus and rhinovirus^43, 44^. However, viruses provide a much simpler template structure than ribosomes as their surface is comprised of regular repeating protein units. Our demonstration of the applicability of MIPs to recover ribonucleoprotein complexes such as the ribosome thus fundamentally extends the scope for MIP application. Besides profiling of translation, we propose that the technology could be extended to other RNPs, such as microRNA-induced silencing complexes^11^ for detection of miRNA-targets; complementing current efforts establishing single-cell transcriptome and proteome analysis^45, 46^.

One of the common obstacles with the MIP technology is the complete removal of the template from the imprint. In this regard, it is possible that extensive washing of the granulated MIPs with physiological buffers already removed a large fraction of template ribosomes as only ~5% of initially applied ribosomes where detectable in the Rt-MIPs (Fig. 3). Whereas some of the ribosomes used for imprinting may have been lost during sieving (typically 5–10%), a majority of template ribosomes may have been bound with low affinity and therefore could be removed from the polymer with physiological buffers. This is not unexpected regarding the large and complex structure of the ribosome, which could form many weak interactions with the polymer at different sites. Conversely, the remaining fraction of template ribosomes may have established high-affinity binding interfaces with the polymer matrix, requiring the implementation of harsh procedures for removal from MIPs. Ultimately, the template ribosomes could be effectively removed (~99.9%), which is in line or even superior to those commonly achieved in the field^32^. Nevertheless, even small residual amounts of template could impose problems for measurements at analytical scale. One strategy to resolve this issue was framed towards testing the selective rebinding of other similarly sized template proteins^34^. This becomes even more possible on the larger macromolecular scale (>100 kDa) where the template behaves more like a particle and it’s selective recognition relies less on individual chemical anchor/docking points (between template and MIP) but is instead dominated by docking based on molecular shape and size^47^. In this line, our data suggests that human R-MIPs can be used to detect specific ribosome-associated mRNAs of related mammalian species, such as simian and mouse. Since mRNAs from different species can be distinguished by RT-PCR with species-specific primers or upon sequencing, we propose that human R-MIPs could provide a valuable tool to access the translation of few specialised cells from these species.

At this stage, there are many possibilities for further optimisation of R-MIPs. In the course of our experiments, we tested different compositions of PAA for polymerisation and the choice of sieve size to granulate the MIPs. Regarding the latter, we found that the optimal sieve size was at 150 μm, compared to smaller sieves of 75 μm and 35 μm, for which we experienced increased non-specific binding of ribosomes to the NIP control (unpublished observations). The former revealed that a mixture of 6% (w/v) acrylamide monomer and 10% (w/w) MBAm cross-linker was optimal, as other concentrations affected template ribosome elution or increased background with NIP controls (unpublished results). One specific area for improvement relates to the seemingly low recovery of ribosome-bound mRNAs from extracts with R-MIPs (between 5–10%; Fig. 4), while it was reported that protein MIPs could recover up to 50% of the initial protein used for imprinting (*e.g*. BSA^32^). We believe that the seemingly low recovery of ribosomes and associated mRNAs is mainly due to technical rather than biological reasons as the mRNAs under investigation are substantially associated with polysomes for translation (65% of yeast actin mRNA^48^; 40% of mouse *eEF2* mRNA^49^; Fig. 4a). The low recovery may relate to the presence of a large fraction of low-affinity binding sites on the polymer, resulting in the removal of most ribosomes upon washing with physiological buffers, which would be in line with our observations made in regard of the recovery of ribosomal templates from granulated Rt-MIPs (see above, Fig. 3). It suggests potential for further improvement to increase the fraction of high-affinity binding sites for ribosomes. Overall, the empirical testing of additional chemical variables *e.g*. functional monomers (in terms of varying hydrophobicity), cross-linker and buffer composition; as well as alternative MIP formats, such as hierarchical MIPs on columns^36^ or attachment of MIP particles to physical supports, such as glass slides or multi-well plates^34, 50^ could further improve the suitability of MIPs towards translatome analysis.

In conclusion, we believe that R-MIPs could eventually become a valuable tool for the study of translation, especially in cases where only small amounts of tissues or cells (*e.g*. stem cells) are available. Since the MIP technology is rather cost effective, it may open the door for integration of translational profiling in high-throughput applications for drug screening and diagnostics; greatly supporting the study of translation and its regulation in health and disease.

## Methods

### Reagents, plasmids and cells

Chemicals and oligonucleotide primers were purchased from Sigma-Aldrich, unless stated otherwise. All reagents/buffers were prepared in RNase-free water and filter sterilised at 0.2 µm. Plasmid pRPS6-GFP was a kind gift of Prof F. Loreni^40^, pGL3 was purchased from Invitrogen (#E1741). HeLaS3 and C8-D1A cells were purchased from ATCC, and cultured in Dulbecco’s Modified Eagle’s Medium (DMEM) supplemented with 5% fetal bovine serum (FBS). Simian VK219 cells (African green monkey, *Chlorocebus aethiops*, kidney epithelial cells latently infected with KSHV and expressing GFP from the EF-1α promoter, as a marker of latent infection^38^) were cultured in Minimum Essential Medium Eagle (Sigma #M2279) supplemented with 10% FBS (Gibco), 2.2 g/L NaHCO_3_, 5 μg/ml puromycin, and 10,000 U/ml penicillin-streptomycin (Life Technologies). The cells were grown in dishes in a humidified incubator at 37 °C in 5% CO_2_. *Saccharomyces cerevisiae* strain BY4741 (MAT**a** *his3*Δ*1 leu2*Δ*0 met15*Δ*0 ura3*Δ*0*) was grown in yeast-peptone-dextrose (YPD) media at 30 °C.

### Purification of ribosome template used for imprinting

HeLa cell cytoplasmic extract (~23 mg/ml) was purchased from IpraCell (Belgium) supplied in 10 mM Hepes-KOH (pH 7.5), 0.5 mM Mg(OAc)_2_, 10 mM KOAc, 0.5 mM DTT. The extracts were supplemented with 1 × complete protease inhibitor (Roche #11836170001) and stored in aliquots at −80 °C. The extract was diluted to 2.3 mg/ml (protein concentration) in 10 ml ribosome buffer (20 mM Tris-HCl, pH 7.5, 150 mM NaCl, 5 mM MgCl_2_, 0.5 mM DTT, 0.5% Triton X-100, 0.2 mg/ml heparin, 1 Units (U)/ml DNase I, 1 × complete protease inhibitor) and centrifuged twice at 18,500 × *g* for 10 min at 4 °C to pellet mitochondria. 10 U of RNase ONE (Promega #M4261) was added to the cleared extract and incubated for 2 min on ice, and the partial RNA digest was terminated by addition of 100 U RNasin (Promega #N2615). Furthermore, 4 M KCl was added drop-wise to a final concentration of 0.5 M to dissociate ribosome-bound proteins. The extract was layered onto a 16 ml sucrose cushion in Ti70 tubes comprised of ribosome buffer supplemented with 20% sucrose, 20 U/ml RNasin. The samples were centrifuged at 100,000 × *g* for 2 h at 4 °C to pellet ribosomes. The supernatant was removed, and 300 µl of ribosome storage (RS) buffer (20 mM Tris-HCl, pH 7.5, 150 mM NaCl, 5 mM MgCl_2_, 0.2 mg/ml heparin) was added to the pellet on ice, and the pellet was gently resuspended upon stirring with a small magnet and increasing speed for a total of 90 min. The re-dissolved ribosomes (~2 µg/µl protein) were aliquoted, snap frozen in liquid nitrogen (LN_2_) and stored at −80 °C.

### Purification of 40S and 60S ribosomal subunits

HeLa cytoplasmic extracts were supplied as described above in ribosome buffer supplied with 0.5 M KCl and centrifuged twice at 18,500 × *g* for 10 min at 4 °C to pellet mitochondria. The supernatant was transferred into a Ti45 tube and centrifuged at 44,000 × *g* for 4 h at 4 °C. The pellet was resuspended in 5 ml ribosome resuspension (RRS) buffer (20 mM Tris-HCl, pH 7.5, 100 mM KCl, 0.25 M sucrose, 2 mM DTT) over 2 h upon gentle stirring at 4 °C and centrifuged at 12,000 × *g* for 5 min at 4 °C to remove non-soluble material. Samples were diluted to an optical density (OD) at 260 nm = 100. Then, 1 mM puromycin was added and incubated for 10 min at 4 °C and for 10 min at 37 °C. 4 M KCl was added drop-wise to a final concentration of 0.5 M, and the extract was layered on top of a linear 10–30% sucrose gradient and centrifuged at 40,000 × *g* for 17 h at 4 °C in a SW28 rotor. Following centrifugation, 1 ml fractions of the gradient were collected manually, and fractions containing the 40S and 60S subunits were identified by agarose gel electrophoresis to detect the 18S and 28S rRNA, respectively. The corresponding fractions were pooled and centrifuged at 34,000 × *g* for 17 h at 4 °C. The pellets containing 40S or 60S subunits were gently resuspended in 500 μl RRS buffer, aliquoted, snap frozen in LN_2_ and stored at −80 °C.

### Polymerisation of acrylamide around ribosomes to generate R-MIPs

MIPs were typically prepared to 50 µl in 1.5 ml lo-bind tubes (Eppendorf #Z666505) on ice. The final MIPs contained 6% total acrylamides (w/v), of which 10% was MBAm and 90% AA. Therefore, 2.7 mg AA and 0.3 mg MBAm were combined in RS buffer, and purified human ribosomes were added to 0.75 µg/µl and gently mixed by pipetting. For control NIPs, RS buffer without ribosomes was added to AA monomers. 0.2% (w/v) APS and 0.1% (w/v) TEMED were added to the reactions, mixed and polymerized under gaseous N_2_ for 10 min on ice, and for a further 10 min with the tube lid closed, resulting in gel formation. Following complete polymerisation as determined by visual inspection (ribosome/co-monomer solutions turn from colourless to semi-opaque after polymerisation), gels were manually passed through a 150 µm mesh (Endecotts Ltd) and collected. The gel fragments were washed three times with 100 µl ice cold RS buffer and collected by centrifugation at 3,500 × *g* for 1 min at 4 °C between washes using an Eppendorf Mini-Spin Plus centrifuge.

### Removal of template ribosomes from the hydrogel

Hydrogels were resuspended in 100 µl alkaline hydrolysis solution (150 mM NaOH, 150 mM EDTA) and incubated at 37 °C for 60 min with orbital shaking at 650 rpm. The reaction was neutralised by addition of 150 mM HCl. The hydrogels were subsequently placed on ice and washed three times with ice cold wash buffer (RS buffer without heparin). The gel was collected between the washes by centrifugation at 3,500 × *g* for 1 min at 4 °C. Gels were then resuspended in 100 µl wash buffer supplemented with 100 µg/ml proteinase K (Promega #V302B), 2.5 mM CaCl_2_, 2.5% glycerol and incubated for ~16 h at 4 °C on an orbital shaker at 650 rpm. 5 mM phenylmethylsulfonyl fluoride (PMSF) was added to stop the reaction and gels incubated for 5 min on ice. Gels were finally washed three times with ice-cold wash buffer. R-MIPs were stored at 4 °C in 100 µl wash buffer and used within one week.

### Capture of extract derived ribosomes with R-MIPs

To ensure heterogeneity of MIP fragments across experimental replicates, the 50 µl hydrogels were combined together using low-bind tips (Elkay #AER-5REF-S96) and equilibrated in reload (RL) buffer (20 mM Tris-HCl, pH 7.5, 150 mM NaCl, 5 mM MgCl_2_, 0.1 mg/ml CHX, 0.1% Triton-X-100, 100 U/ml RNasin, protease inhibitor) then collected by centrifugation (3,500 × *g*, 1 min, 4 °C). The combined MIPs were split on ice into 50 µl aliquots in protein lo-bind tubes and extract corresponding to 1,000 cells was added (~200 ng of total protein/ 6 ng total RNA of extracts derived from HeLa, simian and mouse cells; 6 ng of RNA from yeast extracts; and 6 ng of mouse total RNA as controls). Thereby, extracts were diluted to the desired concentration in 100 µl RL buffer, and applied to the MIP or NIP or a sample kept as input. The tubes were flicked to mix, and then shaken at 650 rpm for 45 min at 4 °C. Gels were collected by centrifugation, and washed three times in RL buffer, frozen in LN_2_, and stored at −80 °C.

### Recovery of RNAs/proteins bound to R-MIPs

Hydrogels were resuspended in 100 µl ice-cold extraction buffer (10% glycerol, 20 mM Tris-HCl 7.5, 150 mM NaCl, 5 mM MgCl_2_, 100 U/ml RNasin, 1 × complete protease inhibitor) and combined with 100 µl of glass beads (0.5 mm, Stratech Scientific Ltd. # 11079105-BSP) and disrupted in a Tissue Lyser (Qiagen, #85300) for 5 cycles, each 2 min at 30 Hz at 4 °C, with 1 min rests between cycles. To collect the soluble fraction, the tube was pierced with a needle, placed in a larger tube, and centrifuged at 500 × *g* for 30 s at 4 °C. For analysis of proteins, half of the R-MIP/ NIP eluate (~50 µl) was concentrated to 10 µl using YM10 spin columns (Merck Millipore #MRCPRT010) and combined with 10 µl 2 × Laemmli sample buffer containing 5% β-mercaptoethanol (BioRad). To assess the RNA, 100 pg of *LysA* RNA (ATCC #87482) was added prior to RNA isolation. To remove the abundant transfected plasmid DNA, samples containing GFP and luc reporter RNAs were additionally digested with 2 U BbvI (NEB) for 30 min at 37 °C. Total RNA was purified with the MicroRNA prep kit (Zymo Research #R1061) including on-column digest with 5 µl DNase I in 35 µl RDD buffer (Qiagen #79254) for 15 min at 37 °C, which was repeated twice by reapplication of the flow-through. RNA was finally eluted from columns with RNase-free water.

### *In vitro* transcription of luc reporter

Luc was amplified from pGL3 (Invitrogen #E1741) by PCR with primers Luc-FwdT7 and Luc-RevT7 as follows: 1 × GoTaqGreen Master mix (Promega #M7122), 0.5 µM T7 Luc-FwdT7, 0.5 µM Luc-RevT7, 100 ng pGL3 were combined in a volume of 20 µl with cycling conditions 95 °C for 5 min, 30 cycles of [95 °C for 15 s, 62 °C for 30 s, 72 °C for 30 s], and 72 °C for 5 min. PCR products were resolved on 1% agarose gel, extracted with phenol-chloroform and precipitated with EtOH. Transcription was performed with the mMESSAGE mMACHINE T7 transcription kit (Ambion #AM1344) as per the manufacturer’s instructions, using 1 µg of DNA template. The reaction was terminated by DNase I treatment, and RNA was polyadenylated with Poly(A) Tailing Kit (Ambion #AM1350) as per the manufacturer’s instructions. RNA was purified using standard phenol-chloroform extraction, precipitated with EtOH and resuspended in RNase-free water. The integrity of the RNA was controlled on a 1% agarose gel.

### *In vitro* translation assays

The rabbit reticulocyte *in vitro* translation assay system (Promega #L4151) was used to monitor translation activity of purified human ribosomes. Reactions were assembled in triplicates in round-bottom 96-well plates. To determine the minimum concentration of CHX to inhibit translation activity of the reticulocyte lysate, serial dilutions of CHX and luciferase (*Luc*) reporter RNA were performed with each batch of reticulocyte lysate (data not shown). Samples were assembled on ice containing 15 µM hemin (in DMSO; Sigma #51280), 50 µg/ml creatine phosphokinase (Merck Millipore #2384), 10 mM creatine phosphate (Merck Millipore #2380), 1 mg/ml yeast tRNA (Sigma #R4752), 2 µl amino acids mix (Promega #L4461), 0.5 µl RNasin (Promega #N2615), 10 µl reticulocyte lysate (Promega #L4151), and 0.5 µM CHX (Sigma #C1988) and incubated at 30 °C for 10 min to inhibit endogenous ribosomes of the lysate; and then placed back on ice. Purified human ribosomes (2 µg, 4 µg or 8 µg of protein) provided in RS buffer and 100 ng of *Luc* reporter mRNA (see above) were added to a final volume of 20 µl; and reactions were incubated at 30 °C for 1 h. To measure luc activity, 5 µl of each reaction was added to 25 µl LarI (Promega #E1500), vortexed for 5 s, and bioluminescence was read for 10 s on a luminometer (Jade). Three independent experiments were performed and bioluminescence for each experiment was measured in triplicate.

### Transient transfections and cell treatment

HeLa were seeded in 6-well plates at 0.4 × 10^6^ cells per well in 2 ml DMEM and FBS. After ~16 h, cells were co-transfected with 250 ng pRPS6-GFP and 2.5 µg pGL3 and 7 µl of Lipofectamine 2000 (Invitrogen #11668027). After 8 h, the media was changed to remove the transfection reagent, and cells were further incubated for 48 h before harvesting. For the serum starved condition (=starved), cells were treated with trypsin to transfer from the 6-well plate onto a 6 cm^2^ dish, and seeded in DMEM without FBS for 2 h to adhere before harvesting. For stimulation of cells with serum (=refed), 10% FBS was added to starved cells and incubated for a further 2 h before harvesting. HEK293, C8-D1A and VK219 cells were seeded onto 10 cm^2^ dishes and harvested at ~80% confluence.

### Lysis of mammalian cells

Cell culture dishes were transferred onto ice, media removed, and cells gently rinsed in ice cold PBS. PBS was removed by aspiration and 1 ml of freshly prepared ice-cold polysome lysis buffer (20 mM Tris-HCl, pH 7.5, 150 mM NaCl, 5 mM MgCl_2_, 0.5% Triton X-100, 0.1% sodium deoxycholate, 0.1 mg/ml CHX, 0.5 mM DTT, 100 U/ml RNasin (Promega #N2615), 10 U/ml DNase I (Promega #M6101), 1 × complete protease inhibitor (Roche #11836170001)) was added directly per 15 cm^2^ dish. Cells were scraped from the dish and collected in a microtube. Lysates were centrifuged at 13,000 × *g* for 5 min at 4 °C to pellet nuclei, and the supernatant transferred to a fresh tube. Unless being used immediately, the extracts were snap frozen in LN_2_ and stored at −80 °C.

### Culture and lysis of yeast *S. cerevisiae* cells

Yeast cells were grown in YPD to mid-log phase (OD_600nm_ ~0.6) and harvested by centrifugation. Cells were resuspended in 1 ml polysome lysis buffer and 500 µl of glass beads (0.5 mm, Stratech Scientific Limited #11079105-BSP) were added and tubes vortexed four times for 20 s with 90 s rests on ice in between. The lysate was cleared by three subsequent centrifugations at 2,600 × g, 8,600 × *g*, and 13,400 × *g*, each for 5 min at 4 °C and stored at −80 °C. Total RNA was isolated with the ZR RNA MicroPrep Kit (Zymo #R1061).

### Sucrose density fractionation

The HeLa cell extract prepared from starved and refed cells were layered on top of a linear 15–50% sucrose gradient prepared in 20 mM Tris-HCl, pH 7.5, 150 mM NaCl, 10 mM MgCl_2_, 0.1 mg/ml CHX and 0.2 mg/ml heparin. The samples were centrifuged at 100,000 × *g* for 2 h at 4 °C in a SW41 swinging bucket rotor, and 1 ml fractions of the gradient were collected while continuously recording the absorbance at 254 nm (A_254_) with a flow cell UV detector (ISCO). 100 pg of LysA RNA (ATCC #87482) was added to each fraction and RNA was subsequently isolated by addition of 3 M guanidine hydrochloride and 50% ethanol and overnight incubation at −20 °C. The RNA was pelleted by centrifugation at 16,000 × *g* for 60 min at 4 °C, resuspended in water and subjected to a second precipitation with 2.5 M LiCl at −20 °C overnight to remove residual heparin. After centrifugation, the RNA pellet was washed with 75% ethanol, dried and resuspended in RNase-free water. RNA was treated with DNase (TURBO DNA-free, Ambion #1907) prior to reverse transcription (RT).

### SDS-PAA Gels and immunoblotting

Proteins were separated on a 15% SDS-PAA gel by electrophoresis and stained with colloidal coomassie for the visualisation of proteins. For immunoblot analysis, proteins were separated on SDS-PAA gels and transferred to a polyvinylidene difluoride (PVDF) membrane (ThermoScientific). Membranes were blocked in PBS containing 0.1% Tween-20 and 5% BSA, and probed with designated antibodies and horseradish peroxidase (HRP)-coupled secondary antibodies. The blots were developed with the Immobilon Western Chemiluminescent HRP substrate (Milipore) and recorded with a FluorChem (Alpha Innotech). The following antibodies were used: mouse anti-GAPDH (1:1,000; Genetex, GTX627408), mouse anti-RPS12^51^ (1:1,000; clone #1B10; gift of S. Volarevic), mouse anti-RPL13 (1:1,000; Abcam, ab58323), mouse anti-RPS6 (1:1,000; gift of S. Volarevic), rabbit anti-RPL26 (1:1,000; Cell Signalling, 5400), mouse anti-β-actin (1:2,000; Sigma, A1978), HRP-conjugated sheep anti-mouse IgG (1:5,000; Amersham, NXA931) and HRP-conjugated donkey anti-rabbit (1:5,000; Amersham, NA9340V).

**Oligonucleotide primer sequences**

Luc-T7Fwd: 5′-GGTTAGCAGCGCTAATACGACTCACTATAGGGTAAAGCCACCATGG-3′

Luc-T7Rev: 5′-GTATCTTATCATGTCTGCTCGAAGCGG-3′

18S rRNA Fwd: 5′-AAACGGCTACCACATCCAAG-3′

18S rRNA Rev: 5′-CAATTACAGGGCCTCGAAAG-3′

28S rRNA Fwd: 5′-CAAAGCGGGTGGTAAACTCC-3′

28S rRNA Rev: 5′-CTCTTAACGGTTTCACGCCC-3′

LysA Fwd: 5′-CTGAAAGCACAGGTGGCATA-3′

LysA Rev: 5′-AGCTCTCTCCGGATACGACA-3′

GFP Fwd: 5′-GACCACATGAAGCAGCACG-3′

GFP Rev: 5′-GGTCTTGTAGTTGCCGTCG-3′

Luc Fwd: 5′-TCATGGATTCTAAAACGGATTACC-3′

Luc Rev: 5′-CGAAGGACTCTGGCACAAA-3′

eEF2 Mus Fwd: 5′-CATGGCCAAGTGGAGTTCCC-3′

eEF2 Mus Rev: 5′-CCGGGCTGCAAGTCTAAGG-3′

GAPDH Mus Fwd: 5′- GAGCATCTCCCTCACAATTTCC-3′

GAPDH Mus Rev: 5′-TGGTATTCAAGAGAGTAGGGAGG-3′

Actin Sc Fwd: 5′-GTCTGGATTGGTGGTTCTATC-3′

Actin Sc Rev: 5′-GGACCACTTTCGTCGTATTC-3′

Tdh1 Fwd: 5′-CCAAGAAGGTTGTCATCACTG -3′

Tdh1 Rev: 5′-GTACAAGAAGCGTTGGAGAC -3′

### RT-PCR and agarose gel electrophoresis

RT was performed using a mixture of oligo(dT) and random primers according to the manufacturer’s instructions (Primer Design RT-nanoscript 2–150) for 2 h at 42 °C. 20 µl PCR reactions were performed with 1 µl of cDNA, 1 × GoTaqGreen Master Mix (Promega #M7122), 0.5 µM of respective forward (Fwd) and reverse (Rev) primers for 2 min at 95 °C, 35 cycles of [95 °C for 30 s, 59 °C for 30 s, 72 °C for 30 s], and 72 °C for 5 min. The products were resolved on a 2% agarose gel supplemented with PeqGreen stain (VWR #PEQL37–5000) and visualised with UV light. Band intensities were quantified with ImageJ software.

### qPCR quantification

Quantitative PCR was carried out with the QuantiTect SYBR Green PCR Kit (Qiagen #204143) using gene-specific primers (see above). 20 µl reactions contained the SYBRGreen master mix, 1 µl of cDNA, and 0.5 µM of each primer. The cycling conditions were 15 min at 95 °C followed by 40 cycles of [15 s at 95 °C, 15 s at 59 °C and 30 s at 72 °C] with data collection at the extension stage. A melt curve was routinely run to ensure primer specificity.

Data were analysed with the “delta delta cycling threshold” (ΔΔCt) method^52^. To calculate the amount of remaining ribosome template in the R-MIP, we first performed in-sample normalisation of 18S rRNA to the spike-in control RNA (LysA). Of note, 18S rRNA was considered as a surrogate for the presence of ribosomes. We further calculated the relative amount of retained 18S rRNA in the R-MIP compared to amounts used for imprinting (input; Fig. 3).

To calculate the relative amount of respective mRNA recovered with MIP/NIPs shown in Fig. 4a, we first performed an in-sample normalisation to *LysA* spike-in control RNA, and calculated levels of mRNAs relative to levels in input extracts applied to the MIP/NIP. To determine IFs (Fig. 4b), the enrichment of a specified RNA (normalised ΔΔCt) with MIPs was divided through respective enrichment (norm. ΔΔCt) with NIP.

To calculate the amount of *RPS6-GF* *P* relative to the reference *Luc* mRNAs captured by the MIP and NIPs in starved and refed HeLa cells (Fig. 5b), we first performed an in-sample normalisation to the *LysA* spiked-in control RNA, and to total RNA levels of extract (input) used to load the R-MIPs/NIPs. The ratio of *RPS6-GFP* to the *Luc* reference was then calculated for each sample, and compared pairwise between refed and the starved samples across three biological replicates *e.g*. ratio of refed MIP1 to starved MIP1, 2, and 3. Fractions 5–12 of polysomes were pooled for likewise analysis to calculate relative changes of *RPS6-GFP* to *Luc* in refed *vs*. starved cells (Fig. 5b).

### Data Availability

The datasets generated during and/or analysed during the current study are available from the corresponding authors on reasonable request.

## Electronic supplementary material

Supplementary Information
