# Supplementary material for: Generation of ribosome imprinted polymers for sensitive detection of translational responses
Source: Sci Rep. 2017 Jul 26;7:6542. doi: 10.1038/s41598-017-06970-x (PMC5529568; doi:10.1038/s41598-017-06970-x)
Supplement: Supplementary file 1 — Supplementary Information [file 41598_2017_6970_MOESM1_ESM.pdf]

Supplementary Figures

Generation of Ribosome Imprinted Polymers for Sensitive Detection of Translational Responses

Helen A. King, Hazim F. El-Sharif, Ana M. Matia-González, Valentina Iadevaia, Adeola Fowotade, Subrayal M. Reddy, André P. Gerber

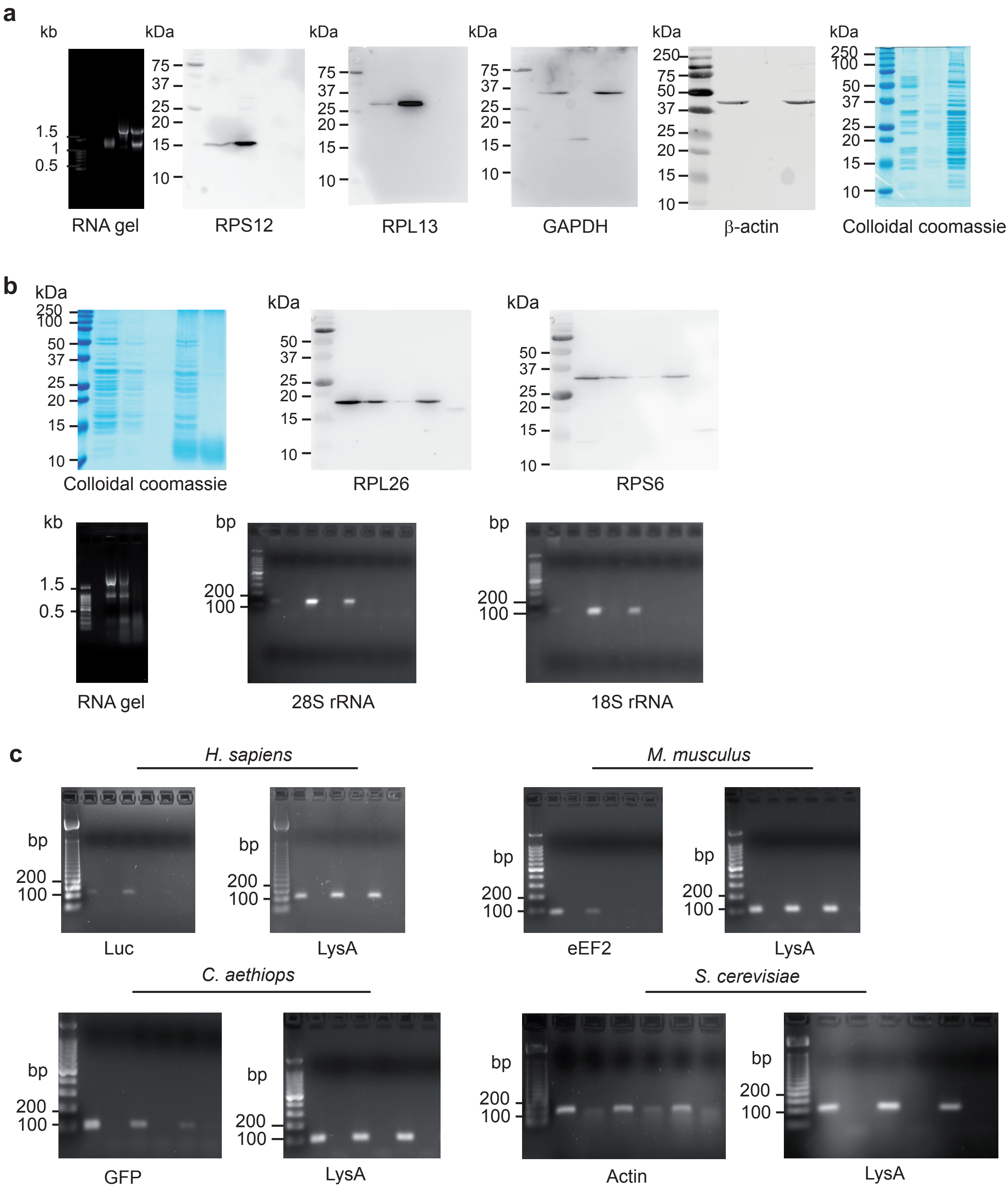

Figure S1. Uncropped gels and immunoblots from Fig.2 (**a**), Fig.3 (**b**) and Fig. 4 (**c**).

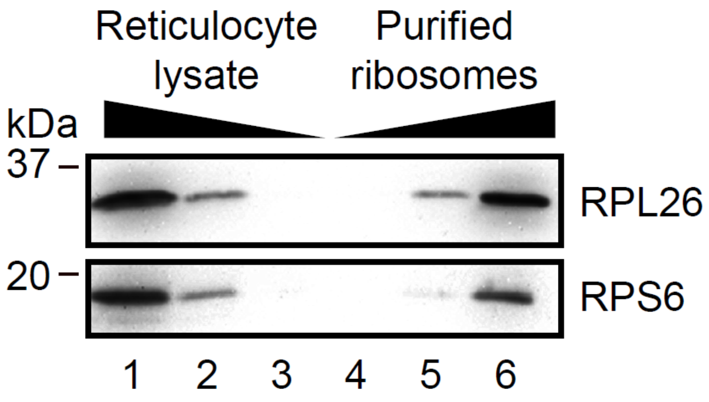

Figure S2. Comparing the amounts of RPs in rabbit reticulocyte lysate with that of purified human ribosomes. An immunoblot probed with antibodies detecting RPL26 and RPS6 is shown. Numbered lanes refer to the following: 1, 10 % of the reticulocyte lysate used in the assay; 2, 2% reticulocyte lysate; 3, 0.4% reticulocyte lysate; 4, 0.032 µg of purified human ribosomes; 5, 0.16 µg purified ribosomes; 6, 0.8 µg purified ribosomes. The amounts of purified human ribosomes was determined with the BCA assay (Pierce) taking BSA as a reference standard.

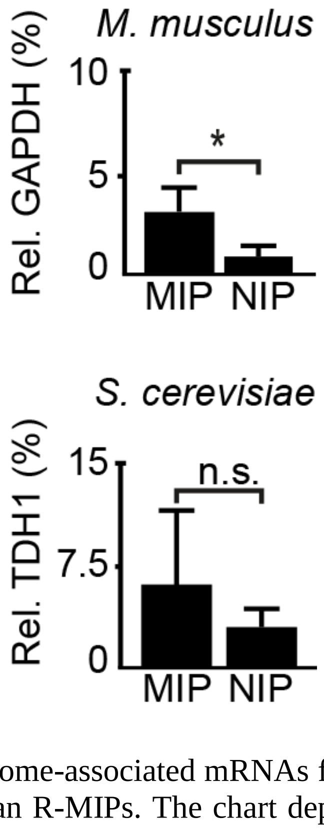

Figure S3. Recovery of ribosome-associated mRNAs from cellular extracts of closely related eukaryotic species with human R-MIPs. The chart depicts the relative recovery of indicated mRNAs with MIPs or NIPs as compared to the input extract (100%). RNA was quantified by RT-qPCR with the ΔΔCt method and normalised to LysA (see Methods). SEMs are shown as bars; *M. musculus*, n = 5, endogenous GAPDH mRNA in C8D1A cells; *S. cerevisiae*, n = 3, TDH1 mRNA in yeast. *, $P < 0.05$, two-tailed homoscedastic student's t-test.

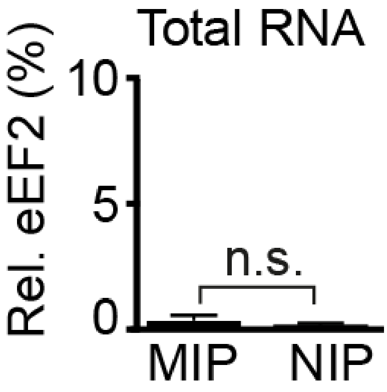

Figure S4. Purified RNA does not bind to the MIP. MIPs or NIPs were reloaded with total RNA isolated from 1,000 C8D1A mouse cells. Mouse *eEF2* mRNA was quantified with RT-qPCR with mouse specific primers. The amount of *eEF2* mRNA detected in the MIP and NIP is shown relative to the input. Error bars represent SEM, n = 3. No significance was found with a two-tailed homoscedastic student's *t*-test.
